# Surgical anatomy and clinical variation of the left colonic artery in laparoscopic anterior rectal resection

**DOI:** 10.3389/fsurg.2023.1190259

**Published:** 2024-01-09

**Authors:** Jiajun Li, Xiaofang Zhao, Bo Yi, Chuanchuan Fu, Peipei Xu, Chao Chen, Bin Zhao, Yangchun Zheng

**Affiliations:** ^1^ Department of Gastrointestinal Surgery, 416 Hospital of Nuclear Industry, Second Affiliated Hospital of Chengdu Medical College, Chengdu, China; ^2^ Department of Gastrointestinal Surgery, Sichuan Cancer Hospital, Chengdu, China; ^3^ Department of Operating Room, Sichuan Cancer Hospital, Chengdu, China

**Keywords:** colorectal cancer, anterior rectal resection, left colonic artery, anatomy, variation

## Abstract

**Objectives:**

This study aims to investigate the surgical anatomy and clinical variation of the left colonic artery (LCA) during laparoscopic anterior rectal resection.

**Methods:**

We conducted a retrospective analysis of 87 patients diagnosed with colorectal cancer who underwent laparoscopic anterior rectal resection with preserved LCA at the Department of Gastroenterology, Sichuan Cancer Hospital, between March 2018 and April 2022, aiming to observe the emanation location, anatomical typing, and travel trajectory of the LCA, as well as its relationship with the inferior mesenteric vein (IMV).

**Results:**

In all observed cases, we observed that the LCA emanated from the left side of the inferior mesenteric artery (IMA), and the average distance from the root of the IMA to the emanation of the LCA was approximately 3.5 ± 1.1 cm. Specifically, 35 of these cases had the LCA branching from the IMA alone (Type I, 40.2%),16 cases had the LCA cotruncating with the sigmoid artery (SA) (Type II, 18.4%), 30 cases had the LCA cotruncating with the superior rectal artery (SRA) and SA (Type III, 34.5%), and six cases had the LCA cotruncating with four or more branches of the SRA and SA (Type IV, 6.9%). No LCA agenesis cases were found in this group. In addition, we also observed the occurrence of LCA alignment. Specifically, there were 25 cases where the LCA crossed the IMV in a diagonal upward direction (Type A, 28.7%), 36 cases where the LCA crossed the IMV in an upward arched manner (Type B, 41.4%), 18 cases where the LCA crossed the IMV in a vertical outward direction (Type C, 20.7%), and eight cases where the LCA crossed the IMV in a diagonal downward manner (Type D, 9.2%). Among them, two cases developed anastomotic fistula, one case had chyle leakage 1 week after surgery, and four cases experienced urinary retention; all of the patients successfully recovered and were discharged after receiving conservative treatment.

**Conclusion:**

The anatomy and variation of the LCA can be clearly and accurately observed during laparoscopic surgery. Understanding the type and variation of the LCA helps to dissect the vessels in the IMA region during surgery, particularly in cases when the LCA is preserved, and reduce the incidence of vascular injury and its complications.

## Introduction

Preserving the left colonic artery (LCA) during laparoscopic anterior rectal resection can improve blood supply to the proximal intestine of the anastomosis. LCA preservation is particularly important in patients undergoing low or ultralow anterior resection, helping to reduce the incidence of postoperative anastomotic fistula (1). Increasing clinical evidence shows that thorough lymph node dissection in the inferior mesenteric artery (D3) at the base of the inferior mesenteric artery (IMA), while preserving the LCA, will not affect the effect of tumour radical treatment or affect the long-term postoperative survival of patients (2). However, due to common anatomic variations, complex classification, and the course of the LCA, the surgical operation of preserving LCA in anterior rectalectomy is difficult. This research examines surgical videos of 77 patients with colorectal cancer undergoing laparoscopic prerectal resection with preserved LCA. The study focuses on observing the location, anatomical classification, course of the LCA, and its relationship with the inferior mesenteric vein (IMV). Furthermore, the surgical anatomy and clinical variation of LCA were discussed to provide information for the anatomy of IMA region-related blood vessels during surgery, particularly to provide more references for the operation of preserving LCA in anterior rectalectomy.

## Data and methods

### General information

A retrospective analysis was conducted on 87 patients who underwent laparoscopic anterior rectal resection with preserved LCA at the Gastrointestinal Surgery Department of Sichuan Cancer Hospital between March 2018 and April 2022. In total, 46 patients were males and 41 were females, and the sample included 78 cases of rectal cancer and nine cases of terminal sigmoid carcinoma. The mean age was 59.6 ± 13.2 years old, the mean height was 161.6 ± 9.2 cm, the mean weight was 57.9 ± 9.9 kg, and the mean body mass index (BMI) was 22.2 ± 3.2 kg/m^2^.

### Surgical methods

Following the administration of general anaesthesia, intubation, disinfecting, and draping, the patients were placed in the modified lithotomy position, and the pneumoperitoneum and the operation channel were established by the traditional five-hole method. The 10-mm trocar above the umbilicus was the observation hole, and the main operation hole was the 12-mm trocar positioned at a point located one-third of the line between the umbilicus and the right anterior superior iliac spine. A 5-mm trocar was inserted at three points, respectively. The three holes were located at the intersection of the right midclavicular line and the plain umbilical line, the intersection of the left midclavicular line and the intersection of the left midclavicular line, and the junction of the navel and the left anterior superior iliac spine and the left midclavicular line, which were used as the auxiliary operation holes and assistant operation holes.

Upon accessing the abdominal cavity, a thorough exploration was conducted, and the middle approach was employed to dissociate the rectum and sigmoid colon. The assistant lifted the upper rectum and the mesum of the sigmoid to maintain a certain tension. The surgeon made an incision at the mesum of the right root of the sigmoid in front of the sacral promontory and entered the left Toldt's space, extending upwards through the space until the IMA root. The vascular sheath of the IMA was incised at the root, an intrathecal dissociation was conducted caudally along the IMA, and vascular skeletonisation was performed to expose the IMA up to the starting point of the LCA. The distance between the IMA root and the LCA emission site was measured intraoperatively. Anatomic dissociation was performed laterally along the course of the LCA, and the origin of the LCA to its junction with the inferior mesenteric vein was exposed. The relationship between the LCA and IMV was identified, and the IMV was further dissociated cephalally. The surgery proceeded with caution to avoid injury to the LCA or IMV, and fat and lymphoid tissue (lymph nodes in 253 groups) were removed and cleaned around the IMA root and between the IMA, LCA, and IMV. The IMV was detached at the IMA root plane. The IMA was disconnected following the emission of the LCA by the IMA.

In accordance with the principle of total mesorectal excision (TME), the dissociation of mesorectal excision was conducted downwards along the vascularless space between the visceral and parietal layers of the pelvic fascia. The low rectal cancer was isolated to the pelvic floor and surgically closed 2 cm distal to the tumour. The tumours in the middle and upper rectum and sigmoid colon were isolated to 5 cm distal to the tumour and closed at 5 cm distal to the tumour. The proximal bowel duct was separated approximately 10–15 cm above the tumour. If the patient is eligible for natural orifice specimen extraction surgery (NOSES), the tumour specimen is removed through the anus (3). If the patient is unsuitable for NOSES surgery, an auxiliary incision of approximately 5 cm was made in the left lower abdomen to remove the specimen. An end-to-end sigmoidorectal anastomosis was performed for intestinal reconstruction using a disposable tube stapler inserted through the anus. For ISR patients with very low tumour location, manual sutures can be used for a sigmoidic-anal end-to-end anastomosis.

**Figure 1:**
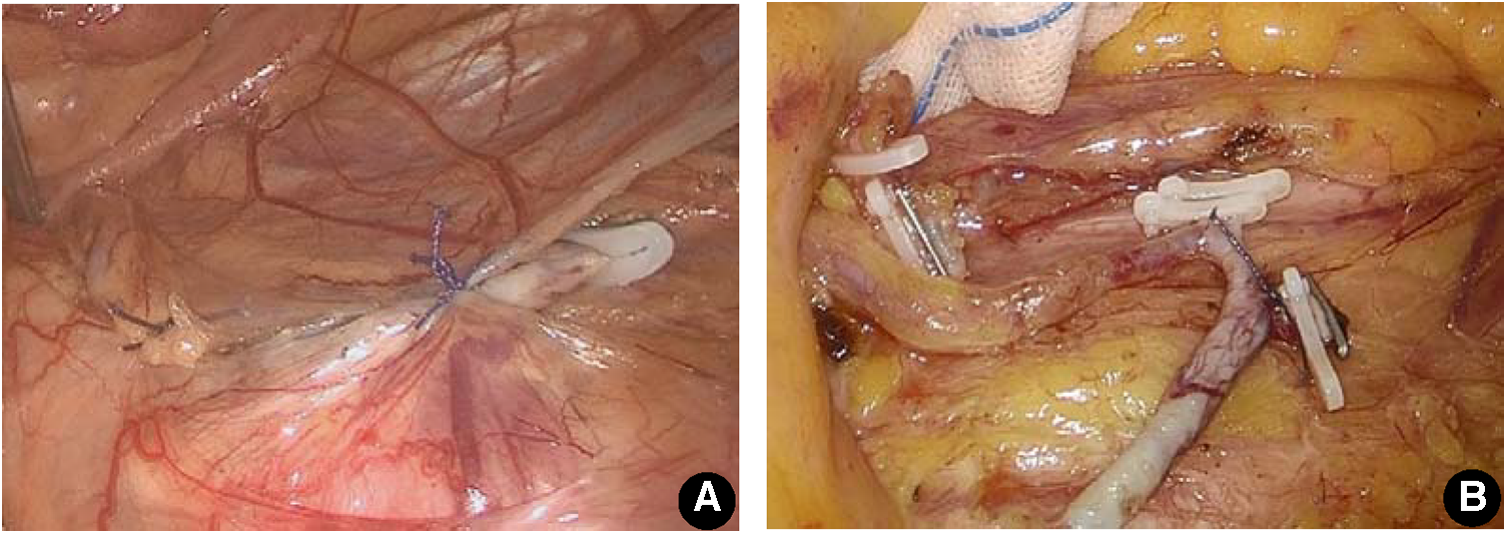
Treatment of the hemiloop formed by the IMA root, LCA, and IMV after lymph node dissection in 253 groups. (**A**) Suture the posterior peritoneum of the IMA root with the mesangium of the descending colon and cover the vascular ring behind the mesocolon. (**B**) The site of IMA ligature was sutured onto the posterior peritoneum to avoid excessive dissociation of the hemicyclin.

Prior to the end of the operation, a total of 253 groups of lymph nodes were dissected, and a vascular half-ring with a larger diameter surrounded by IMA root, LCA and IMV might be formed. The vascular half-ring should be covered by using conventional sutures to attach the descending mesocolon and posterior peritoneum, or the IMA dissection and ligature below LCA could be sutured and fixed on the posterior peritoneum using 3-0 absorbable thread. Avoid the risk of intestinal herniation due to excessive dissociation of the hemicycle (see Figure 1).

### Statistical analysis

SPSS21.0 statistical analysis software was used for data processing to test the normality of the sample data. The measurement data obeyed a normal distribution and are described as the mean ± standard deviation (x ± s). Counting information is expressed as quantity and percentages.

## Results

### The originating location of the LCA

In this group of 87 patients, the LCA was emitted from the left side of the IMA in all cases, and the average distance between the root of the IMA and the origin of the LCA was approximately 3.5 ± 1.1 cm.

### Classification of LCA

**Figure 2:**
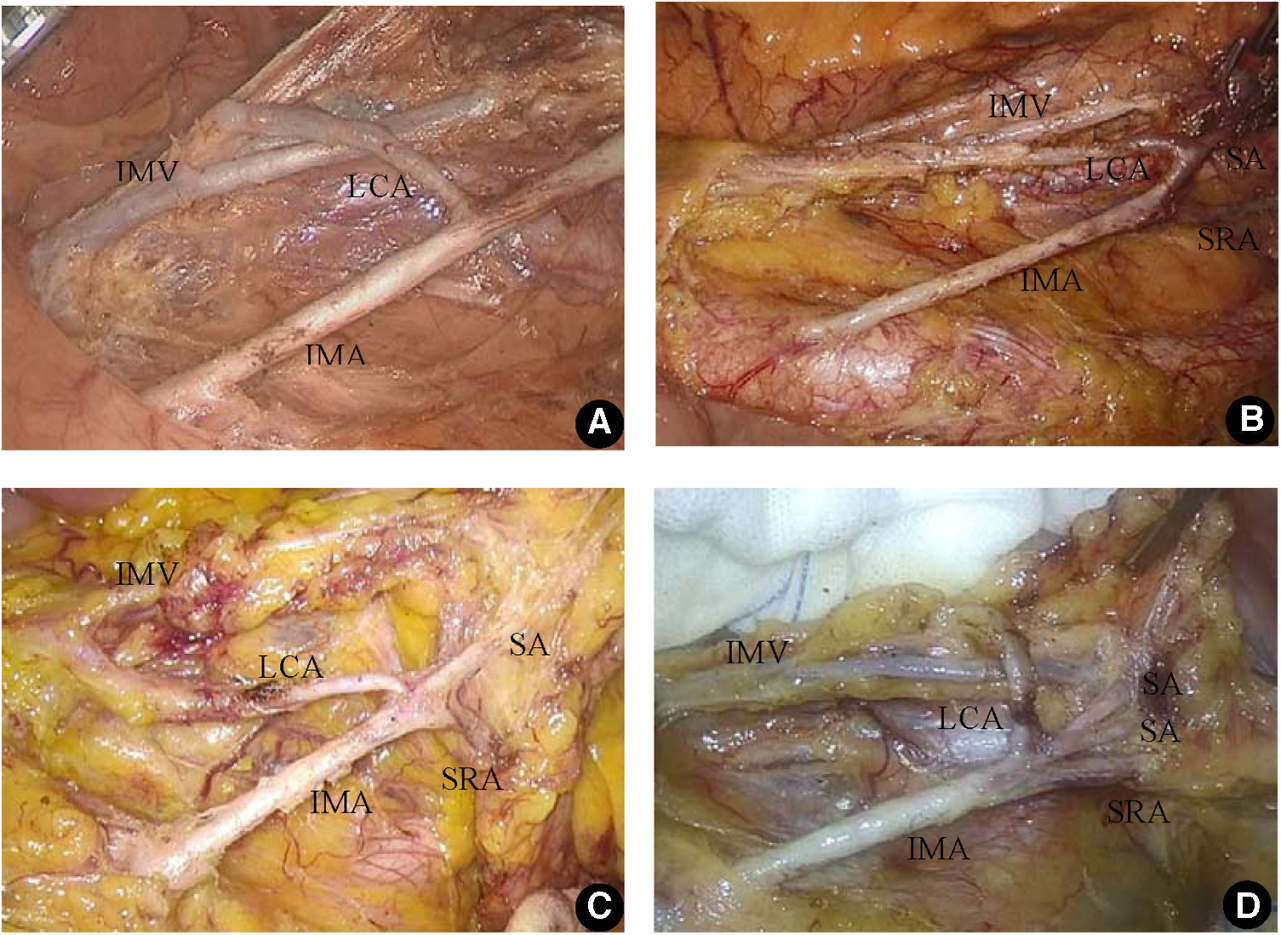
Branch type of the inferior mesenteric artery. (**A**) The LCA was issued from the IMA alone as the first branch (Type I). (**B**) The SA emanated from the LCA, and the LCA and SA stem together (Type II). (**C**) The LCA, SRA, and SA simultaneously stem from the IMA (Type III). (**D**) The LCA stem had four or more branches of the SRA and SA and emanated from the IMA simultaneously (Type IV).

When the rectal artery of the LCA was in conjunction with its sigmoid artery (SA) and superior rectal artery (SRA), it was divided into the following four types: In Type I, the LCA was issued from the IMA alone as the first branch, and the LCA and SA were not costemmed in 35 cases (40.2%). In Type II, the SA emanated from the LCA, and the LCA and SA stem together in 16 cases (18.4%). For Type III, the LCA, SRA, and SA simultaneously stem from the IMA in 30 cases (34.5%). For Type IV, the LCA stem had four or more branches of the SRA and SA and emanated from the IMA simultaneously in six cases (6.9%). No cases of LCA deficiency were found in this group (see Figure 2).

### Relationship between LCA and IMV

**Figure 3:**
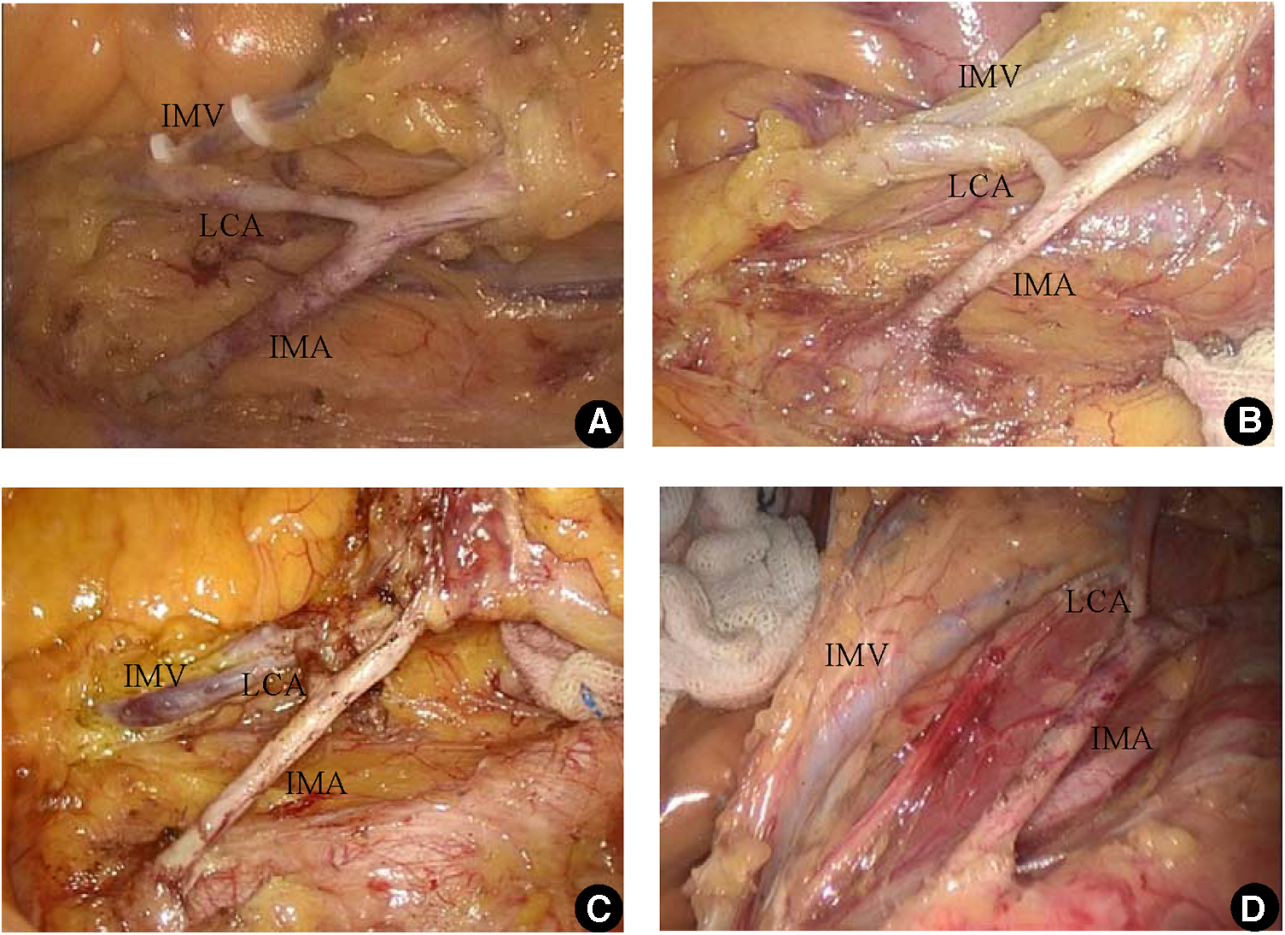
The relationship between LCA and IMV. (**A**) The LCA crossed the IMV diagonally (Type A); (**B**) the LCA arcuated upwards and crossed the IMV (Type B); (**C**) the LCA crossed vertically with the IMV (Type C); (**D**) the LCA crossed the IMV diagonally downwards (Type D).

The anatomic position relationship between the LCA and IMV was classified into four types: Type A, LCA crossed the IMV diagonally, with a total of 25 cases (28.7%); Type B, the LCA arcuated upwards and crossed the IMV, with a total of 36 cases (41.4%); Type C, the LCA crossed vertically with the IMV, with 18 cases (20.7%); and Type D, the LCA crossed the IMV diagonally downwards in eight cases (9.2%) (See Figure 3).

### Postoperative complications

Two patients developed fistula drainage, resembling faecal discharge, on postoperative days 3 and 5, which were confirmed as anastomotic fistulas through CT imaging. They were managed with fasting, anal dilation, and drainage tube irrigation, and eventually discharged after recovery.

One patient experienced lymphatic fistula on postoperative day 6, which gradually ceased with measures such as fasting, parenteral nutrition, and the administration of somatostatin.

Four patients experienced urinary retention, but they recovered and were discharged following conservative treatment, including the use of urinary catheterisation.

## Discussion

Due to the advancements in surgical technology and the maturity of endoscopy technology, tumour surgery has developed from the traditional pursuit of radical treatment to the direction of ensuring radical treatment that is as minimally invasive as possible and preserves function to the maximum extent. Surgery with functional preservation primarily focuses on three aspects: organ, nerve, and blood vessel preservation (4, 5).

The blood supply of the proximal anastomotic bowel in laparoscopic anterior rectal resection is derived from two main sources: (1) the blood supply of the left branch of the middle colic artery through the marginal vascular arch and (2) the arterial blood supply of the LCA. During the operation, when the high-level IMA was severed, the blood supply from the LCA was severed simultaneously, and the blood supply to the proximal intestinal duct of the anastomosis could only rely on the blood delivered by the left branch of the middle colic artery through the marginal artery, which led to a decrease in blood flow to the anastomosis. For patients with incomplete or absent anastomosis of the vascular network at the edge of the splenic curvature of the colon (the key point of Griffiths), relying only on the marginal artery will also obstruct blood flow in the proximal intestinal canal and may even result in intestinal ischaemic necrosis. Retaining the LCA not only helps improve the blood supply to the anastomosis, but also avoids the potential risk associated with the defect of the vascular network at the edge of the key points of Griffiths. The problem of intestinal blood supply is more prominent particularly for patients undergoing low and ultralow anterior rectal excision due to the longer intestinal segment of the proximal sigmoid that needs to be preserved (6–9). Anastomotic fistula is one of the most serious complications following anterior resection of the rectum, which can significantly increase the surgical duration, hospital costs, and even necessitate a secondary surgery, causing greater trauma and suffering for the patients. In this study, two cases of anastomotic fistula occurred among the patients but were successfully treated conservatively, and they both recovered well and were discharged smoothly. Currently, multiple studies have confirmed, using Doppler ultrasound, that preserving the left colonic artery significantly enhances blood supply to the proximal colon tube of the anastomosis (10). In addition, many scholars have shown through comparative studies that preserving the left colonic artery can reduce the risk of anastomotic fistula (11, 12).

LCA retention during laparoscopic anterior rectal resection is argued to not be conducive to a total resection of the lymph nodes at the IMA root, which affects the radical treatment of tumours. However, in recent years, increasing clinical evidence has shown that a thorough dissection of 253 lymph nodes does not reduce the radical treatment of the tumour nor does it affect the long-term survival of patients (1, 2). Another factor that affects the enthusiasm of surgeons to retain the LCA is that the anatomy of the LCA considerably varies, and the surgical operation to retain the LCA is of high technical difficulty and will prolong the operation time (13, 14). However, with the accumulation of experience of surgeons, the improvement of techniques, and the upgrading of endoscopic instruments and equipment, this problem is gradually being overcome.

The authors' experience shows that the key to preserving the LCA is to open the vascular sheath of the IMA, and an intrathecal anatomy was performed along the vessel. An intrathecal dissection not only helps to thoroughly clean the lipolymphatic tissue around the IMA but also helps to find the root of the LCA more clearly. This process is especially advantageous for patients with a high BMI and thick mesocolon. In this study, the LCA, SRA, and SA simultaneously costemmed from the IMA in 42.6% of the cases (Type Ⅲ and Type Ⅳ). Without intravaginal dissection, blood vessels were prone to damage in these cases, which results in accidental bleeding while searching for the LCA; consequently, the LCA cannot be retained.

The method to classify the LCA proposed by the authors according to the cooccurrence of LCA, SA, and SRA is mainly based on the practical perspective of guiding surgical operation. This typing method and that proposed by Yada et al. (15) for LCA variant typing is the added Type Ⅳ, which involves the special type of LCA codrying with four or more SRA and SA. In the classification method proposed by Murono et al. (16), LCA was absent for Type IV. Since no LCA-absent cases were found in this group, the LCA-absent cases were not classified. The authors suggest that the absence of an LCA should be classified as Type 0 for ease of understanding and memory.

Based on the classification method, Type I accounted for 40.2% of the cases, and dissociation was the simplest due to the absence of codrying between LCA and SA. The key is to avoid damaging the trunk of the LCA. Type Ⅱ accounted for 18.4% of the cases. Attention should be given to the presence of branch vessels below the LCA during the dissociation process. Type III and Type IV surgeries are the most difficult. Because the LCA is codried with SA and SRA and simultaneously originates from the IMA, multiple vessels are gathered in the common opening. Therefore, we must be careful during LCA root dissociation and exposure, ensuring not only the complete removal of lymphatic tissue but also the prevention of any damage to blood vessels.

The authors divided the LCA into four types according to the anatomic relationship between the LCA and IMV for the purpose of guiding surgical operations. Type A and Type B accounted for 70.1% of the total, and the LCA line was upwards. Therefore, when dissociating LCA, the emphasis was on tracing and revealing the LCA upwards; Type C accounts for 20.7% of cases, for which the LCA crosses horizontally with IMV vertically. Therefore, the distance between the root of this type of LCA and IMV is the shortest. When dissociating, caution should be taken to avoid IMV damage. Type D accounts for 9.2% of the cases. Since LCA is inclined downwards, it is easily mistaken for the absence of an LCA if the LCA is not revealed throughout. Therefore, in the case of oblique downwards movement of the first IMA branch during the operation and a suspected absence of the LCA, the vascular anatomy must be thoroughly analysed to trace the lateral IMV to the vascular arch at the edge of the colon to determine that it does not serve as a blood donor for the descending colon (17–19). The LCA was present in all cases in this group.

At the same time, the authors noted that the IMA root, LCA, and IMV will form a semicircle after the LCA is fully exposed and the lower part of the IMA is severed. If the diameter of the semicircle is larger than the diameter of the small intestine, the small intestine may herniate into the vascular ring due to peristalsis, thus forming an internal abdominal hernia and loop-closing ileus, which may tear the vascular ring and have fatal consequences. In this group of patients, small intestine herniation into the vascular ring occurred during the operation. Fortunately, timely detection and corresponding prevention were performed. The method to prevent LCA intravascular hernia is to suture the posterior peritoneum of the IMA root with the mesangium of the descending colon and cover the vascular ring behind the mesocolon. For cases with mesocolon defects that are too large to be sutured to the posterior peritoneum of the IMA root, the site of IMA ligature can also be sutured onto the posterior peritoneum with 3-0 absorbable thread, which can also avoid the effect of intestinal herniation into the vascular ring of the LCA.

In summary, this paper reviewed the surgical videos of 77 patients undergoing laparoscopic anterior rectal resection with preserved LCA, discussed the branch types, shape characteristics, and variation of LCA, and summarised the relationship between LCA classification and IMV for the purpose of facilitating surgical operation. The authors believe that the anatomical data obtained from *in vivo* observations during laparoscopy is more closely related to the clinical reality, and that understanding and familiarity with the anatomy and variation of LCA will certainly contribute to the anatomy of the vessels related to the IMA region in surgical procedures, particularly in the operation of preserving LCA during anterior rectal resection.

## Data Availability

The original contributions presented in the study are included in the article/Supplementary Material, further inquiries can be directed to the corresponding author.
